# Threshold effects of sleep duration and cognitive function in older adults with BMI ≥ 25 kg/m^2^

**DOI:** 10.3389/fnagi.2024.1529639

**Published:** 2025-01-07

**Authors:** Kunyu Qiu, Yilei Liu, Chengwei Hu, Jie Gu, Yanyan Huang

**Affiliations:** ^1^Shanghai Putuo District Changzheng Town Community Health Service Center, Shanghai, China; ^2^Department of General Medicine, Huashan Hospital, Fudan University, Shanghai, China

**Keywords:** cognitive function, overweight and obesity, sleep duration, threshold effects, NHANES

## Abstract

**Background:**

It has been demonstrated that older adults’ cognitive capacities can be improved with sleep duration. However, the relationship between overweight, obesity, and cognitive decline remains a subject of debate. The impact of sleep duration on cognitive performance in seniors with a body mass index (BMI) ≥ 25 kg/m^2^ is largely unknown. This makes it an intriguing subject to explore further.

**Methods:**

This study used data from the National Health and Nutrition Examination Survey (NHANES) (2011–2014) with 2,243 participants. Weighted multivariate linear regression and smooth curve fitting were employed to investigate linear and non-linear relationships. A two-part linear regression model was used to determine the threshold effects. Additionally, subgroup analysis and interaction tests were conducted.

**Results:**

Results showed that a negative association was found between sleep duration and scores in the fully adjusted model in the Consortium to Establish a Registry for Alzheimer’s Disease (CERAD) test, the Animal Fluency test (AFT), and the Digit Symbol Substitution test (DSST). A two-piecewise linear regression model was then applied to explore the threshold effect of sleep duration on cognitive performance. When sleep duration was less than 5 and 6 h per day, sleep duration was positively correlated with CERAD test scores [ß (95% CI): 2.11 (1.17, 3.05), *p* < 0.0001], AFT scores [*β* (95% CI): 0.25 (−0.17, 0.67), *p* = 0.2376], and DSST scores [ß (95% CI): 0.49 (−0.57, 1.56), *p* = 0.3654]. However, there was a threshold effect where sleep duration reached the three inflection points.

**Conclusion:**

In overweight and obese older adults, there is a clear inverted U-shaped relationship between sleep duration and cognitive function, with consistent results across different subgroups. Sleep durations of around 5–6 h may help prevent cognitive decline in older adults with a BMI ≥ 25 kg/m^2^.

## 1 Introduction

We are living in an aging world, where a substantial number of people are likely to experience age-related cognitive decline, now one of the leading causes of disability worldwide (Alzheimer's disease facts and figures, 2015; Prince et al., 2015). Globally, dementia currently impacts over 50 million individuals, with projections indicating a threefold increase in prevalence by 2050, primarily driven by an aging population (Nichols et al., 2022). In general, cognitive decline is a significant public health concern that can result in dementia or mild cognitive impairment (Alzheimer's disease facts and figures, 2024; Tolar et al., 2020).

According to earlier research, obesity and being overweight contribute to midlife cognitive decline and dementia, which accounts for one-third of all dementia cases globally (Pedditzi et al., 2016). Some studies have found that obesity and being overweight have been connected to alterations in volumetric cortical and subcortical function (Beyer et al., 2019). These modifications are linked to decreased cognitive function and adjustments to the white matter microstructure (Samara et al., 2019). Working memory (Alarcón et al., 2016), verbal memory, processing speed, fluid intelligence (Spyridaki et al., 2014), and executive function are among the cognitive domains that are impacted (Morys et al., 2021). However, recent studies suggest that being overweight and obese may be beneficial for older adults in late life (Norton et al., 2014). An increasing number of meta-analyses support the concept of the “obesity paradox” (Kim et al., 2020; Talaei et al., 2020), suggesting that obesity and being overweight may have a protective effect against cognitive decline in middle-aged and older adults (Pedditzi et al., 2016). Therefore, the impact of obesity and being overweight on the risk of cognitive impairment or dementia remains a subject of debate (Gustafson, 2015).

Over the past few decades, the prevalence of obesity has risen significantly (Global BMIMC et al., 2016; Lu et al., 2014; Ngandu et al., 2015). Sleep deprivation and narcolepsy have become increasingly prevalent in older adults (Cheng et al., 2021). Sleep deprivation exerts substantial effects on both brain structure and function (Cheng et al., 2021). Numerous studies have demonstrated that poor sleep is associated with an increased risk of dementia (He et al., 2024; Liu et al., 2016; Lee et al., 2024). However, a study from China found that sleep duration has an inverted U-shaped relationship with cognitive scores, with both short and long sleep durations associated with lower cognitive scores (Li et al., 2022). Moreover, regarding nighttime sleep duration, an optimal range of approximately 7–8 h has been associated with a reduced risk of cognitive impairment. Both insufficient and excessive nighttime sleep significantly increases the risk of cognitive decline (Xu et al., 2020).

Limited research has investigated the association between sleep duration and cognitive function in overweight and obese older adults. This cross-sectional study aims to explore the relationship between sleep duration and cognitive function in older adults who are overweight or obese. Using threshold effect analysis, it identifies the optimal nighttime sleep duration for achieving peak cognitive function in this population, intending to provide lifestyle recommendations for dementia prevention among older adults who are overweight or obese in the United States.

## 2 Materials and methods

### 2.1 Study population

This cross-sectional study utilized data from NHANES, a national survey conducted by the National Center for Health Statistics (NCHS) to assess Americans’ health and nutritional conditions. NHANES employed a sophisticated, multistage probability sampling design to obtain a nationally representative sample of the non-institutionalized US population. Participants provided information about demographics, socioeconomic status, and health status through a household interview, whereas mobile examination centers (MECs) handled laboratory and physical assessments.

The NCHS Research Ethics Review Board authorized all NHANES study methods, and all participants provided informed consent. Detailed information about the study design and data can be obtained at www.cdc.gov/nchs/NHANES/.

**Figure 1:**
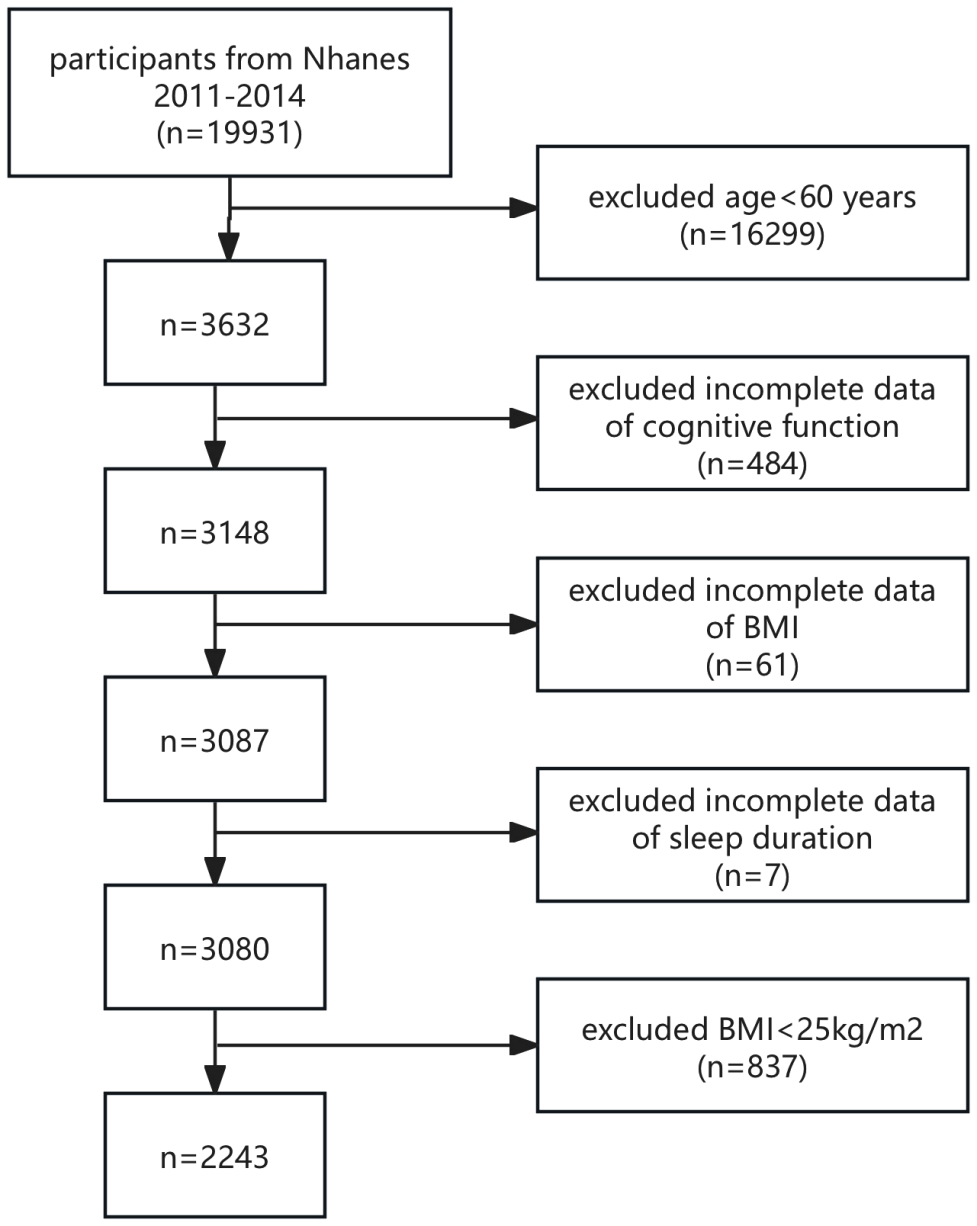
Flowchart of the sample selection from NHANES 2011–2014.

The study population was recruited from NHANES 2011–2014, and all participants with complete CERAD test, AFT, DSST, BMI, and sleep duration data were included in this study. According to the World Health Organization (WHO), overweight is defined as a BMI between 25 and 29.9 kg/m^2^, and obesity is defined as a BMI of 30 kg/m^2^ or higher. Therefore, in this study, we defined overweight and obesity in the study population as a BMI ≥ 25 kg/m^2^. The World Health Organization also classifies those 60 years of age and older as older adults (Beard et al., 2016). Consequently, individuals aged 60 and above were classified as older adults in our research. A total of 19,931 participants were enrolled. After excluding participants with age < 60 years (*n* = 16,299), missing cognitive assessment (*n* = 484), BMI (*n* = 61), and sleep duration (*n* = 7) data, and excluding BMI < 25 kg/m^2^ (*n* = 837), a final total of 2,243 eligible participants were enrolled in this study (Figure 1).

### 2.2 Explanatory variable: sleep duration

Participants’ sleep duration was assessed with the question, “How much sleep (do you/does SP) usually get at night on weekdays or workdays?” Sleep duration ranged from 2 to 11 h, and if participants reported more than 12 h, it was recorded as 12 h, representing total nighttime sleep. Nighttime sleep plays a vital role in brain neurorepair, memory consolidation, metabolic waste clearance, and cognitive function maintenance. This includes synchronization with the circadian rhythm, which regulates different sleep stages like slow-wave and rapid-eye-movement sleep, crucial for brain health. Nighttime sleep also supports cognitive functions by influencing neurotransmitter balance (e.g., dopamine, norepinephrine) and hormones (e.g., growth hormone, cortisol). Furthermore, it minimizes external disruptions, ensuring higher sleep quality (Bubbico et al., 2019; You et al., 2019; Gabelle et al., 2017). In this study, sleep duration was considered a continuous variable.

### 2.3 Outcome variable: cognitive function

The CERAD test evaluates immediate and delayed recall of newly learned verbal information (Fillenbaum et al., 2008). The CERAD test comprises three consecutive learning trials followed by a delayed recall. Participants are asked to read aloud a list of 10 unrelated words in each learning trial. Afterward, they immediately recall as many words as possible. The delayed recall occurs approximately 10 min after the learning trials. Each trial has a maximum score of 10 points, with a total possible score of 40 points, combining the results of the three learning trials and the delayed recall.

The AFT assesses verbal category fluency, which is a measure of executive function, along with other cognitive abilities such as semantic memory and processing speed (Clark et al., 2009). In this task, participants are required to generate as many animal names as possible within a one-minute timeframe, with one point awarded for each correct response.

The DSST is a comprehensive assessment of cognitive functioning involving processing speed, visual scanning, sustained attention, and short-term memory (Casagrande et al., 2021). The test is administered on paper, with a key at the top showing nine numbers paired with unique symbols. Participants are given 2 min to match and copy the corresponding symbols into the 133 boxes that are aligned with the numbers.

### 2.4 Assessment of covariates of interest

According to factors identified in previous research that are associated with sleep duration or cognitive function, this study controlled for several covariates of interest, including sex (male/female), age (years), marriage (married/divorced/widowed/living alone), race (Mexican American/other Hispanic/non-Hispanic white/non-Hispanic black/other race), the income-poverty ratio of family (<1/≥1), education level (less than middle school/less than high school/high school or GED/ college or AA degree/college or higher), smoking status (≥ 100-lifetime cigarettes/ <100-lifetime cigarettes), alcohol intake (≥12 drinks per year/ <12 drinks per year), hypertension (yes/no), hyperlipidemia (yes/no), and diabetes mellitus (yes/no/borderline).

### 2.5 Statistical analysis

In descriptive analyses, continuous variables are summarized by mean and standard error (SE). To examine the association between sleep duration and cognitive test scores in overweight and obese older adults, multivariable regression models were used, accounting for the NHANES complex sampling design (sampling weights). Three different model analyses were performed. The crude model was unadjusted for any covariates, Model 1 adjusted for sex, age, and race, and Model 2 adjusted for sex, age, race, marital status, education level, income-poverty ratio, hypertension, hyperlipidemia, diabetes, smoke status, and alcohol intake status. Generalized additive models (GAM) and smoothed curves were also used to address potential non-linear relationships between sleep duration and cognitive test scores. If a non-linear association was observed, a piecewise linear regression model (segmented regression) was used to fit each interval and calculate the threshold effect. A likelihood ratio test comparing a linear model (non-segmented) with the piecewise linear regression model was conducted to determine the presence of a threshold effect. The inflection point (K) connecting the two segments was determined using the maximum likelihood model and a two-step recursive method. Subgroup analysis was performed using stratified multivariable logistic regression models, with stratification factors including sex (male/female), age (≤69/70–79/>79 years), smoke status (≥100 lifetime cigarettes/<100 lifetime cigarettes), alcohol intake (≥12 drinks per year/<12 drinks per year), hypertension (yes/no), hyperlipidemia (yes/no), and diabetes mellitus (yes/no/borderline). These stratification factors were also treated as potential effect modifiers, and interaction terms were added using likelihood ratio tests to assess heterogeneity in associations across different subgroups. All statistical analyses were performed using the R software (version 4.2) and the EmpowerStats add-in (version 4.2). The threshold for statistical significance was set at a two-tailed *p*-value of 0.05.

## 3 Results

### 3.1 Baseline characteristics

The study population comprised 2,243 individuals aged 60 years or older (including 60 years) with a BMI above the normal range, drawn from NHANES (2011–2014). Of these, 1,095 (48.82%) were male and 1,148 (51.18%) were female. The mean age of the 2,243 participants was 69.35 years, the mean sleep duration was 7.04 h, and the mean values of the three tests related to cognitive function (CERAD test, AFT, and DDST) were 24.38, 16.51, and 45.88, respectively.

Analysis of sleep duration as a continuous variable across different demographic, lifestyle, and health categories revealed significant differences in sleep duration based on age, race, hypertension status, overweight or obesity status, and different CERAD test score ranges. These findings were statistically significant (*p* < 0.05) (Table 1).

**Table 1:** Mean ± Standard error (SE) in sleep duration (hours/day) by level of demographic variables, lifestyle variables influencing sleep duration, and classification of cognitive function scores.

Sleep duration (hours/day)	*N*	Mean ± SE	*p*-value
Sex			0.515
Male	1,095	7.06 ± 1.46	
Female	1,148	7.02 ± 1.51	
Age (years)			<0.001*
<=69	1,212	6.87 ± 1.47	
>69, <=79	688	7.12 ± 1.45	
>79	343	7.46 ± 1.51	
BMI (kg/m^2^)			0.010*
> = 25, <30	1,071	7.12 ± 1.39	
> = 30	1,172	6.96 ± 1.57	
Race			<0.001*
Mexican American	240	7.00 ± 1.46	
Other Hispanic	246	6.80 ± 1.44	
Non-Hispanic White	1,041	7.32 ± 1.38	
Non-Hispanic Black	577	6.70 ± 1.59	
Other Race–Including Multi-Racial	139	6.81 ± 1.59	
Education level			0.182
Less than 9th grade	286	6.85 ± 1.60	
9-11th grade (Includes 12th grade with no diploma)	335	7.11 ± 1.55	
High school graduate/GED or equivalent	524	7.06 ± 1.56	
Some college or AA degree	639	7.00 ± 1.44	
College graduate or above	459	7.12 ± 1.33	
Income-poverty ratio			0.761
<1	365	7.02 ± 1.73	
> = 1	1,697	7.04 ± 1.42	
Alcohol intake			0.153
Alcohol intake > = 12 drinks /year	1,458	7.07 ± 1.47	
Alcohol intake <12 drinks /year	732	6.95 ± 1.50	
Smoke status			0.564
Smoke > = 100 cigarettes /life	1,130	7.07 ± 1.49	
Smoke <100 cigarettes /life	1,113	7.00 ± 1.48	
Diabetes mellitus			0.077
Yes	617	6.96 ± 1.61	
No	1,515	7.08 ± 1.44	
Borderline	111	6.82 ± 1.42	
Hyperlipidemia			0.359
Yes	1,310	7.07 ± 1.45	
No	933	7.00 ± 1.54	
Hypertension			0.002*
Yes	1,518	7.05 ± 1.49	
No	725	7.01 ± 1.46	
Score of the CERAD test			0.007*
<10	65	7.60 ± 2.07	
10–19	436	7.11 ± 1.67	
20–29	1,195	7.02 ± 1.44	
>29	547	6.95 ± 1.33	
Score of the AFT			0.236
<10	198	7.23 ± 1.80	
10–19	1,418	7.01 ± 1.50	
20–29	545	7.00 ± 1.33	
>29	46	6.91 ± 1.17	
Score of the DSST			0.161
<10	24	6.75 ± 1.67	
10–19	104	7.29 ± 1.58	
20–29	270	7.09 ± 1.54	
>29	1732	7.00 ± 1.43	

BMI, body mass index; CERAD, the Consortium to Establish a Registry for Alzheimer’s Disease; AFT, the Animal Fluency test; DSST, the Digit Symbol Substitution test. Significant values (*P* < 0.05) are in bold.

### 3.2 Association between sleep duration and cognitive function in overweight and obese older adults

This study found that in the demographic model (Model 1), each additional hour of sleep was associated with a decrease of 0.22 in CERAD test scores (*β* = −0.22, 95% CI: −0.40, −0.04), a decrease of 0.16 in AFT scores (*β* = −0.16, 95% CI: −0.30, −0.01), and a decrease of 0.60 in DSST scores (*β* = −0.60, 95% CI: −1.04, −0.17).

In the fully adjusted model (Model 2), sleep duration remained negatively associated with CERAD test, AFT, and DSST scores. Each additional hour of sleep was associated with a decrease of 0.25 in CERAD test scores (*β* = −0.25, 95% CI: −0.43, −0.07), a decrease of 0.17 in AFT scores (β = −0.17, 95% CI: −0.32, −0.02), and a decrease of 0.78 in DSST scores (β = −0.78, 95% CI: −1.16, −0.40). All results were statistically significant (*p* < 0.05) (Table 2).

**Table 2:** Association between sleep duration and cognitive function in multiple regression model.

	Crude	Model 1	Model 2
Score of the CERAD test	−0.30(−0.49, −0.11) 0.0021*	−0.22 (−0.40, −0.04) 0.0183*	−0.25 (−0.43, −0.07) 0.0063*
Score of the AFT	−0.10 (−0.25, 0.06) 0.2306	−0.16 (−0.30, −0.01) 0.0353*	−0.17 (−0.32, −0.02) 0.0231*
Score of the DSST	−0.37 (−0.87, 0.13) 0.1493	−0.60 (−1.04, −0.17)0.0067*	−0.78 (−1.16, −0.40) <0.0001*

Crude: Unadjusted. Model 1: Adjusted for sex, race, and age. Model 2: Adjusted for sex, race, age, marital status, income-poverty ratio, education level, smoke status, alcohol intake, hypertension, hyperlipidemia, and diabetes mellitus. Significant values (*p* < 0.05) are in bold.

### 3.3 Subgroup analysis

**Figure 2:**
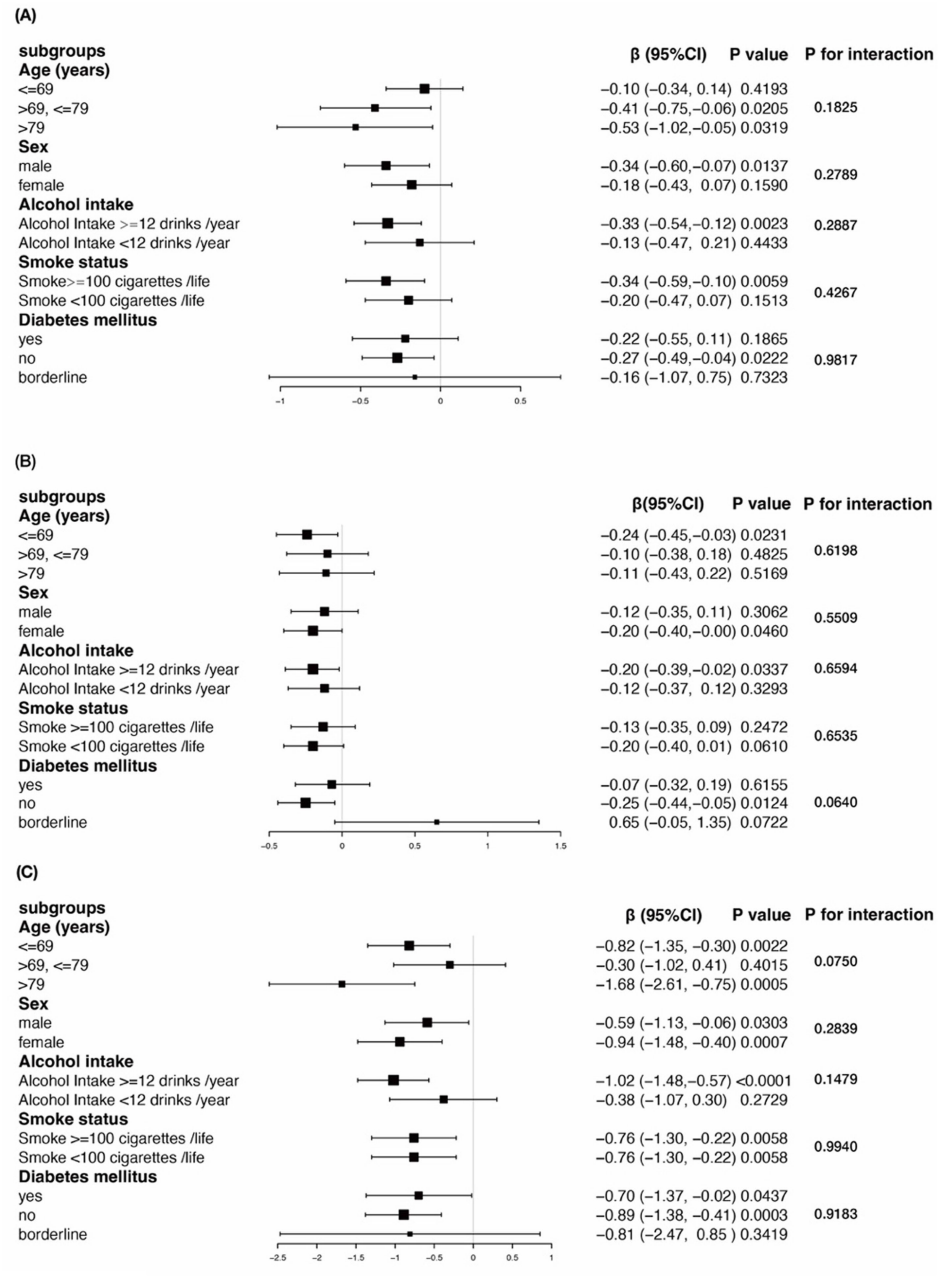
Subgroup analysis for the association between sleep duration and cognitive function in older adults with BMI ≥ 25 kg/m^2^.

Our study indicates a negative correlation between cognitive function and sleep duration. Based on previously identified confounding factors, we further assessed the relationship between sleep duration and cognitive function in both predefined and exploratory subgroups. Stratified analyses and interaction tests were performed as shown in Table 3 and Figure 2 (Association between sleep duration and different cognitive function scores stratified by age, sex, alcohol intake, smoke status, diabetes mellitus. Adjusted for all presented covariates. (A) CERAD test; (B) AFT; (C) DSST.* *p* < 0.05).

**Table 3:** Effect size of sleep duration (hours/day) on cognitive function in prespecified and exploratory subgroups.

Sleep duration (hours/day)			
	**Score of the CERAD test**	**Score of the AFT**	**Score of the DSST**
Age (years)			
<=69	−0.10 (−0.34, 0.14) 0.4193	−0.24 (−0.45, −0.03) 0.0231*	−0.82 (−1.35, −0.30) 0.0022*
>69, <=79	−0.41 (−0.75, −0.06) 0.0205*	−0.10 (−0.38, 0.18) 0.4825	−0.30 (−1.02, 0.41) 0.4015
>79	−0.53 (−1.02, −0.05) 0.0319	−0.11 (−0.43, 0.22) 0.5169	−1.68 (−2.61, −0.75) 0.0005*
P for interaction	0.1825	0.6198	0.0750
Sex			
Male	−0.34 (−0.60, −0.07) 0.0137*	−0.12 (−0.35, 0.11) 0.3062	−0.59 (−1.13, −0.06) 0.0303*
Female	−0.18 (−0.43, 0.07) 0.1590	−0.20 (−0.40, −0.00) 0.0460*	−0.94 (−1.48, −0.40) 0.0007*
*P* for interaction	0.2789	0.5509	0.2839
Alcohol intake			
Alcohol Intake > = 12 drinks /year	−0.33 (−0.54, −0.12) 0.0023*	−0.20 (−0.39, −0.02) 0.0337*	−1.02 (−1.48, −0.57) <0.0001*
Alcohol Intake <12 drinks /year	−0.13 (−0.47, 0.21) 0.4433	−0.12 (−0.37, 0.12) 0.3293	−0.38 (−1.07, 0.30) 0.2729
*P* for interaction	0.2887	0.6594	0.1479
Smoke status			
Smoke > = 100 cigarettes /life	−0.34 (−0.59, −0.10) 0.0059*	−0.13 (−0.35, 0.09) 0.2472	−0.76 (−1.30, −0.22) 0.0058*
Smoke <100 cigarettes /life	−0.20 (−0.47, 0.07) 0.1513	−0.20 (−0.40, 0.01) 0.0610	−0.76 (−1.30, −0.22) 0.0058
*P* for interaction	0.4267	0.6535	0.9940
Diabetes mellitus			
Yes	−0.22 (−0.55, 0.11) 0.1865	−0.07 (−0.32, 0.19) 0.6155	−0.70 (−1.37, −0.02) 0.0437
No	−0.27 (−0.49, −0.04) 0.0222	−0.25 (−0.44, −0.05) 0.0124	−0.89 (−1.38, −0.41) 0.0003
Borderline	−0.16 (−1.07, 0.75) 0.7323	0.65 (−0.05, 1.35) 0.0722	−0.81 (−2.47, 0.85) 0.3419
*P* for interaction	0.9817	0.0640	0.9183

All presented covariates were fully adjusted (as Model 2). Significant values (*p* < 0.05) are in bold.

The results demonstrated that there were no significant interactions between any of the stratifying variables and the relationship between sleep duration and cognitive function. Specifically, the negative association between sleep duration and cognitive test scores in overweight and obese older adults was consistent across all stratified groups (all *p*-values for interaction >0.05).

### 3.4 Non-linear correlation between sleep duration and cognitive function in older adults with BMI ≥ 25 kg/m^2^

**Figure 3:**
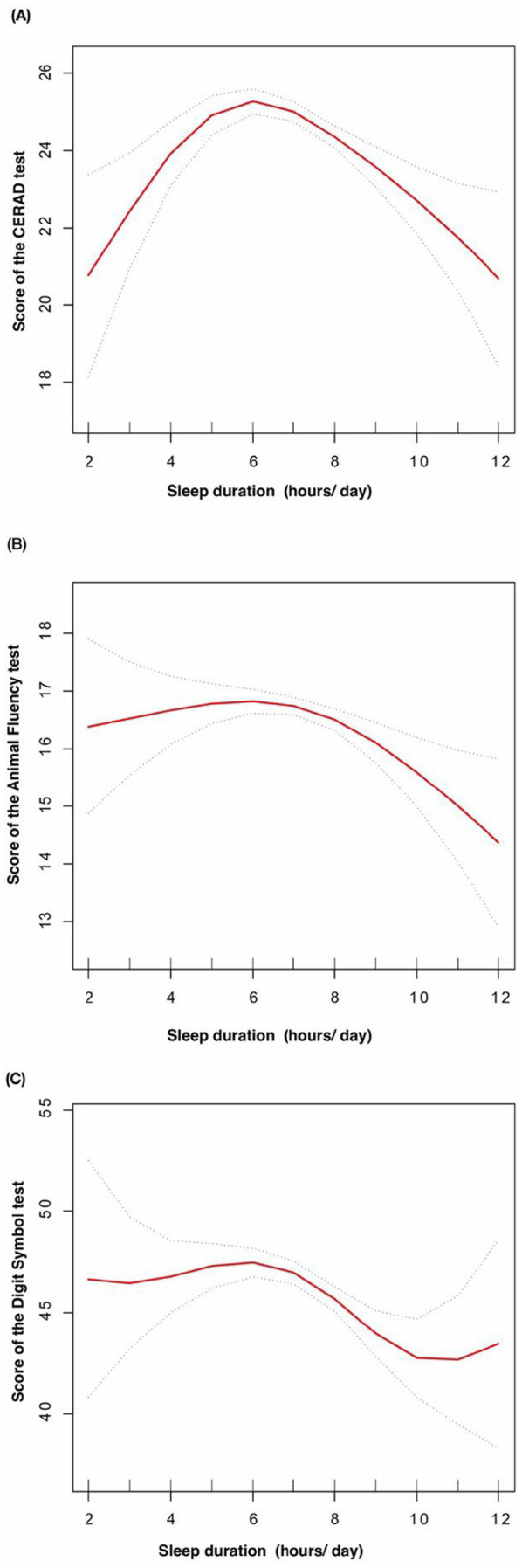
The dose-response relationship between sleep duration with cognitive function in obese elders.

The solid red line represents the smooth curve fit between variables. The dotted line represents the 95% confidence interval for the fit. The dose–response relationship between sleep duration with the score of the CERAD test (A), the score of the Animal Fluency test (B), and the score of the Digit Symbol Substitution test (C) in obese elders (Figure 3).

### 3.5 Threshold effect analysis

After fully adjusting for covariates, we applied a threshold effect model and identified the inflection points (K) for the CERAD test, AFT, and DSST at 5, 6, and 6 h of sleep, respectively. To the left of the inflection point for CERAD test, a positive association between sleep duration and CERAD test was detected (*β* = 2.11, 95% CI: 1.17, 3.05). However, to the right of this point, sleep duration was negatively associated with CERAD test (*β* = −0.51, 95% CI: −0.71, −0.30), with a log-likelihood ratio (LLR) of <0.001.

With regard to AFT, no statistically significant association was observed between sleep duration and the inflection point (*β* = 0.25, 95% CI: −0.17, 0.67). However, a robust inverse correlation was observed on the right side of the inflection point (*β* = −0.32, 95% CI: −0.52, −0.12), with an LLR of 0.0019.

Similarly, no significant association was identified between DSST and sleep duration on the left side of the inflection point (*β* = 0.49, 95% CI: −0.57, 1.56). In contrast, a clear negative association was observed on the right side (*β* = −1.21, 95% CI: −1.71, −0.70), with an LLR of 0.012 (Table 4).

**Table 4:** Threshold effect analysis of sleep duration (hours/day) on cognitive function.

Outcome	Score of the CERAD test	Score of the AFT	Score of the DSST
Fitting by the standard linear Model	−0.25 (−0.43, −0.07) 0.0064*	−0.17 (−0.32, −0.02) 0.0231*	−0.78 (−1.16, −0.40) <0.0001*
Fitting by the two-piecewise linear Model			
Inflection point (K)	5	6	6
<K-segment effect	2.11 (1.17, 3.05) < 0.0001*	0.25 (−0.17, 0.67) 0.2376	0.49 (−0.57, 1.56) 0.3654
>K-segment effect	−0.51 (−0.71, −0.30) <0.0001*	−0.32 (−0.52, −0.12) 0.0019*	−1.21 (−1.71, −0.70) <0.0001*
Log likelihood ratio	<0.001*	0.032*	0.012*

Adjusted for sex, race, age, marital status, income-poverty ratio, education level, smoke status, alcohol intake, hypertension, hyperlipidemia, and diabetes mellitus. CERAD, the Consortium to Establish a Registry for Alzheimer’s Disease; AFT, the Animal Fluency test; DSST, the Digit Symbol Substitution test. Significant values (*p* < 0.05) are in bold.

## 4 Discussion

In this population-based study, a negative correlation was observed between sleep duration and cognitive function in overweight and obese older adults. Furthermore, the relationship between sleep duration and cognitive function was found to be non-linear. Our study’s results indicate a threshold effect between sleep duration and cognitive function in overweight and obese older adults. Specifically, cognitive test scores demonstrated a significant improvement with increasing sleep duration up to a certain point, after which a decline was observed. In particular, a decline in cognitive scores was observed following 5 h of sleep for the CERAD test total score, 6 h for the animal fluency test, and 6 h for the digit symbol substitution test.

The findings of our study indicate an inverted U-shaped relationship between sleep duration and cognitive function in overweight and obese older adults, which is consistent with the results of previous clinical studies conducted in aging populations (Li et al., 2022; Keil et al., 2023; Fjell et al., 2023). Using cohort data from the China Health and Retirement Longitudinal Study revealed that moderate sleep duration, rather than prolonged sleep, was associated with higher cognitive function. A further study based on the UK Biobank dataset provided evidence that both excessive and limited sleep are important risk factors of cognitive impairment in older adults, which aligns with the findings presented here (Yu et al., 2023).

Moreover, a population-based study has corroborated the finding that the optimal duration of sleep for cognitive function is approximately 5–7.5 h, with sleep extending up to 8 h being associated with a decline in cognitive performance (Coulthard and Blackman, 2021). A meta-analysis of nine cohort studies also identified a U-shaped dose–response relationship between sleep duration and the risk of cognitive impairment, with the lowest risk observed at 7–8 h of sleep (Xu et al., 2020).

Our research focuses on globally significant issues such as population aging, overweight and obesity, and the prevention of dementia risk through lifestyle interventions. Earlier studies generally concluded that overweight and obesity has a negative impact on cognitive function. However, recent scholars have introduced the concept of the “obesity paradox” (Kim et al., 2020). In this study, after adjusting for multiple covariates, we found that among overweight and obese older adults, the relationship between sleep duration and cognitive function follows a threshold effect. Specifically, the risk of cognitive decline is lowest when total nighttime sleep duration is around 5–6 h. This conclusion remains robust across different subgroups.

The study results show a reverse U-shaped relationship between sleep duration and cognitive function, which is consistent with other research findings. However, through threshold effect analysis, the optimal sleep duration, compared to 7–8 h, was shortened to 5–6 h, which is an interesting finding. This may be due to the potential protective effects of overweight and obesity on cognitive decline in middle-aged and older adults (Pedditzi et al., 2016), aligning with the concept of the “obesity paradox.”

In addition, several potential mechanisms may explain the observed reverse U-shaped association: sleep is essential for restorative functions and maintaining homeostasis, and prolonged sleep may indicate circadian dysregulation, associated with sleep disorders and cognitive impairment (Devore et al., 2014). Moreover, increased levels of interleukin-6 (IL-6) and C-reactive protein (CRP) have been observed in long sleepers (Benington, 2000), suggesting a link between prolonged sleep, inflammation, and cognitive impairment. The prefrontal cortex, critical for executive function, may be particularly vulnerable to sleep disturbances (Cavaillès et al., 2023) and may be particularly vulnerable to sleep disorders (Thomas et al., 2000; Yaffe et al., 2016).

Short sleep duration may disrupt glymphatic clearance, leading to amyloid-*β* accumulation, associated with neurodegenerative processes in Alzheimer’s disease (Xie et al., 2013). Insufficient sleep has also been associated with an increased risk of cardiovascular disease and related risk factors (Jike et al., 2018; Bock et al., 2022; Itani et al., 2017), and it may also lead to increased inflammation and HPA(Hypothalamic–Pituitary–Adrenal) axis activity (Minkel et al., 2014), which could be another pathway contributing to cognitive impairment, especially in older adults, all of which are linked to cognitive decline and dementia risk (Livingston et al., 2020; Yaffe et al., 2020). Cognitive decline may result from the degradation of neurons that promote wakefulness and sleep (Oh et al., 2019).

Our study holds its own strengths. First, the study utilized a large and representative sample based on the NHANES database. Second, three different models were employed to adjust for potential confounders, enhancing the reliability of our findings. And then, by conducting subgroup analyses, we examined the robustness of the association between sleep duration and cognitive function in overweight and obese older adults across different groups. Finally, our study provides insights into managing sleep duration in overweight and obese populations to maximize cognitive benefits.

However, the results of this study should be interpreted with caution for several limitations. Although the NHANES database is well-suited for cross-sectional studies, further research is needed to elucidate the mechanisms underlying the association between sleep duration and cognitive function in the overweight and obese older adults. While we accounted for several covariates in this study, it is not possible to exclude all potential confounders. Due to the limitation of the self-reported sleep questionnaires from 2011 to 2014, which only included total nighttime sleep duration without data on sleep quality or daytime sleep, this study has certain constraints. Future research should further investigate the relationship between multidimensional aspects of sleep and cognitive abilities.

## Data Availability

The original contributions presented in the study are included in the article/Supplementary material, further inquiries can be directed to the corresponding author/s.
