# Supplementary material for: Threshold effects of sleep duration and cognitive function in older adults with BMI ≥ 25 kg/m2
Source: Front Aging Neurosci. 2025 Jan 7;16:1529639. doi: 10.3389/fnagi.2024.1529639 (PMC11747229; doi:10.3389/fnagi.2024.1529639)

Supplementary Material

**Table 5. Basic characteristics of the study participants.**

| Sleep duration (per day) | <7 hours | 7-9 hours | >9 hours | P-value |
| --- | --- | --- | --- | --- |
| n | 779 | 1366 | 98 |  |
| Gender , n(%) |  |  |  | 0.501 |
| male | 370 (47.50%) | 680 (49.78%) | 45 (45.92%) |  |
| female | 409 (52.50%) | 686 (50.22%) | 53 (54.08%) |  |
| Age (years) | 68.38 ± 6.47 | 69.74 ± 6.78 | 71.59 ± 7.29 | **<0.001*** |
| BMI (kg/m^2^) | 32.23 ± 6.23 | 31.09 ± 5.08 | 31.97 ± 6.37 | **<0.001*** |
| Race , n(%) |  |  |  | **<0.001*** |
| Mexican American | 87 (11.17%) | 147 (10.76%) | 6 (6.12%) |  |
| Other Hispanic | 108 (13.86%) | 129 (9.44%) | 9 (9.18%) |  |
| Non-Hispanic White | 262 (33.63%) | 724 (53.00%) | 55 (56.12%) |  |
| Non-Hispanic Black | 260 (33.38%) | 295 (21.60%) | 22 (22.45%) |  |
| Other Race - Including Multi-Racial | 62 (7.96%) | 71 (5.20%) | 6 (6.12%) |  |
| Education level , n(%) |  |  |  | 0.051 |
| Less than 9th grade | 117 (15.02%) | 157 (11.49%) | 12 (12.24%) |  |
| 9-11th grade  (Includes 12th grade with no diploma) | 112 (14.38%) | 201 (14.71%) | 22 (22.45%) |  |
| High school graduate/GED or equivalent | 184 (23.62%) | 311 (22.77%) | 29 (29.59%) |  |
| Some college or AA degree | 222 (28.50%) | 395 (28.92%) | 22 (22.45%) |  |
| College graduate or above | 144 (18.49%) | 302 (22.11%) | 13 (13.27%) |  |
| Income-poverty ratio | 2.47 ± 1.57 | 2.63 ± 1.60 | 2.03 ± 1.44 | **<0.001*** |
| Sleep hours (per day) | 5.42 ± 0.83 | 7.71 ± 0.66 | 10.41 ± 0.77 | **<0.001*** |
| Alcohol intake , n(%) |  |  |  | 0.278 |
| Alcohol intake ≥12 drinks /year | 494 (64.74%) | 895 (67.09%) | 69 (74.19%) |  |
| Alcohol intake <12 drinks /year | 269 (35.26%) | 439 (32.91%) | 24 (25.81%) |  |
| Smoke status , n(%) |  |  |  | 0.576 |
| Smoke >=100 cigarettes /life | 380 (48.78%) | 695 (50.88%) | 55 (56.12%) |  |
| Smoke <100 cigarettes /life | 399 (51.22%) | 671 (49.12%) | 43 (43.88%) |  |
| Diabetes mellitus , n(%) |  |  |  | **0.005*** |
| yes | 242 (31.07%) | 342 (25.04%) | 33 (33.67%) |  |
| no | 494 (63.41%) | 957 (70.06%) | 64 (65.31%) |  |
| borderline | 43 (5.52%) | 67 (4.90%) | 1 (1.02%) |  |
| hyperlipidemia , n(%) |  |  |  | 0.349 |
| yes | 435 (55.84%) | 819 (59.96%) | 56 (57.14%) |  |
| no | 344 (44.16%) | 547 (40.04%) | 42 (42.86%) |  |
| hypertension , n(%) |  |  |  | 0.124 |
| yes | 532 (68.29%) | 915 (66.98%) | 71 (72.45%) |  |
| no | 247 (31.71%) | 451 (33.02%) | 27 (27.55%) |  |
| Score of the CERAD test | 24.60 ± 6.83 | 24.51 ± 6.77 | 20.88 ± 8.43 | **<0.001*** |
| Score of the AFT | 16.36 ± 5.57 | 16.76 ± 5.51 | 14.21 ± 5.37 | **<0.001*** |
| Score of the DSST | 45.65 ±16.80 | 46.50± 17.37 | 38.15±15.25 | **<0.001*** |

Median ± standard deviation for continuous; n (%) for categorical

Abbreviations: BMI, body mass index; CERAD, the Consortium to Establish a Registry for Alzheimer’s Disease; AFT, the Animal Fluency test; DSST, the Digit Symbol Substitution test.

Significant values (P < 0.05) are in red bold.

**Table 6. Association between sleep duration and cognitive function in multiple regression model.**

|  | Crude | Model 1 | Model 2 |
| --- | --- | --- | --- |
| Score of the CREAD test | -0.30(-0.49, -0.11) **0.0021*** | -0.22 (-0.40, -0.04) **0.0183*** | -0.25 (-0.43, -0.07) **0.0063*** |
| <7 hours | refer | refer | refer |
| 7-9 hours | -0.09 (-0.69, 0.52) 0.7769 | -0.06 (-0.63, 0.51) 0.8298 | -0.33 (-0.89, 0.23) 0.2515 |
| >9 hours | -3.72 (-5.16, -2.28) <0.0001 | -3.27 (-4.61, -1.93) <0.0001 | -2.79 (-4.13, -1.44) <0.0001 |
| P for trend | **0.004*** | **0.007*** | **0.003*** |
| Score of the AFT | -0.10 (-0.25, 0.06) 0.2306 | -0.16 (-0.30, -0.01) **0.0353*** | -0.17 (-0.32, -0.02) **0.0231*** |
| <7 hours | refer | refer | refer |
| 7-9 hours | 0.40 (-0.09, 0.89) 0.1089 | 0.03 (-0.43, 0.49) 0.9093 | -0.15 (-0.62, 0.31) 0.5143 |
| >9 hours | -2.15 (-3.33, -0.97) 0.0004 | -2.19 (-3.29, -1.10) <0.0001 | -1.66 (-2.78, -0.55) 0.0036 |
| P for trend | 0.486 | **0.049*** | **0.047*** |
| Score of the DSST | -0.37 (-0.87, 0.13) 0.1493 | -0.60 (-1.04, -0.17)**0.0067*** | -0.78 (-1.16, -0.40) **<0.0001*** |
| <7 hours | refer | refer | refer |
| 7-9 hours | 0.85 (-0.70, 2.39) 0.2826 | -0.29 (-1.63, 1.04) 0.6660 | -1.22 (-2.38, -0.06) 0.0402 |
| >9 hours | -7.50 (-11.36, -3.64) 0.0001 | -8.50 (-11.80, -5.20) <0.0001 | -6.28 (-9.18, -3.38) <0.0001 |
| P for trend | 0.258 | **0.004*** | **<0.001*** |

Note: Crude: Unadjusted.

Model 1: Adjusted for gender, race, and age.

Model 2: Adjusted for gender, race, age, marital status, income-poverty ratio, education level, smoke status, alcohol intake, hypertension, hyperlipidemia, and diabetes mellitus.

Significant values (P < 0.05) are in red bold.

Figure A and Figure B.


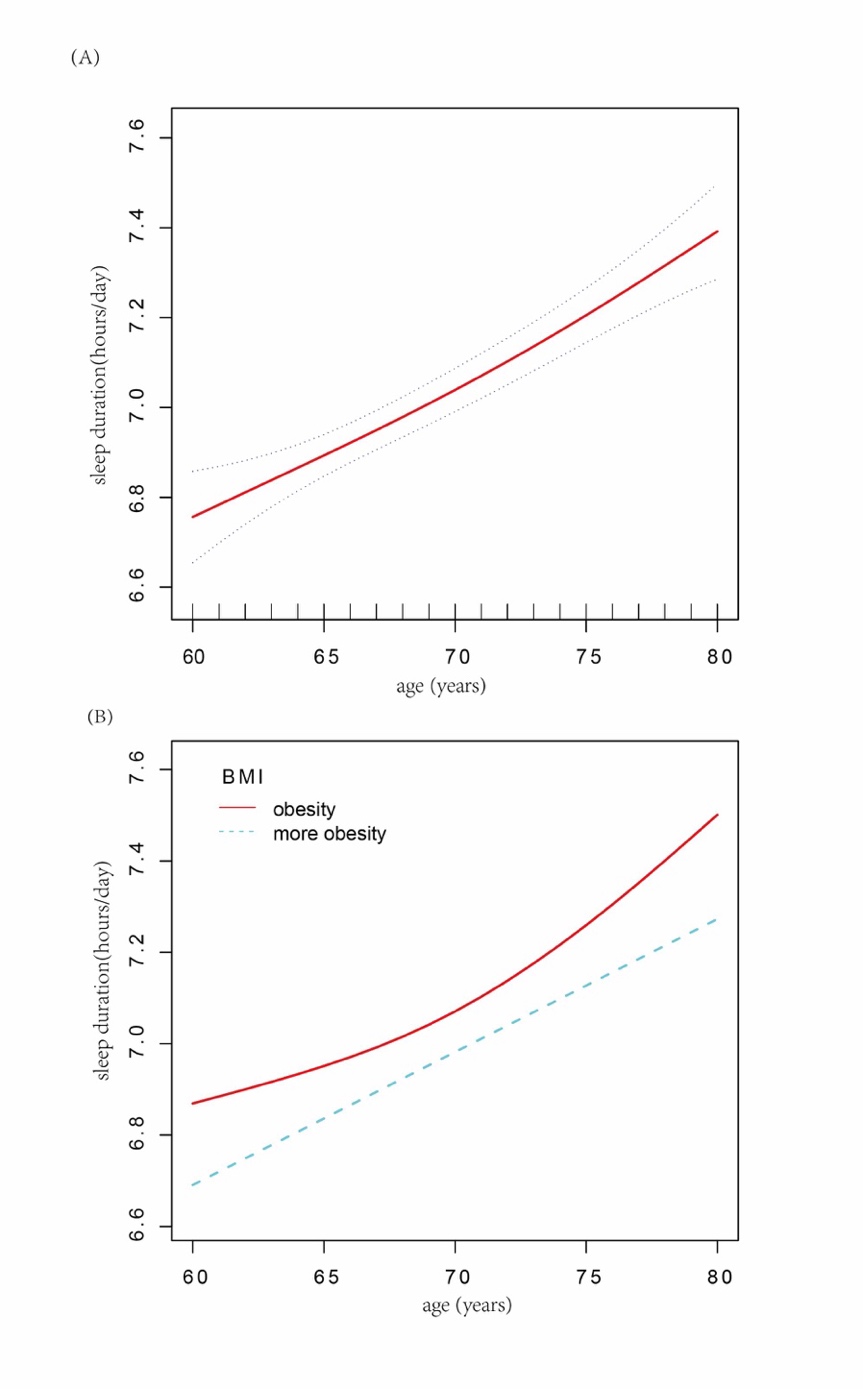

Supplement: Supplementary file 1 [file Table_1.docx]
